# Clonal replacement of epidemic KPC-producing *Klebsiella pneumoniae* in a hospital in China

**DOI:** 10.1186/s12879-017-2467-9

**Published:** 2017-05-23

**Authors:** Yuying Liang, Xiuyun Yin, Lijun Zeng, Shuiping Chen

**Affiliations:** Department of Laboratory Medicine, Affiliated hospital of Academy of Military Medical Sciences, Beijing, People’s Republic of China

**Keywords:** *Klebsiella pneumoniae*, Carbapenemase, Clonal replacement, Epidemiology, Antimicrobial resistance

## Abstract

**Background:**

*Klebsiella pneumoniae* is a frequent nosocomial pathogen causing difficult-to-treat infections worldwide. The prevalence of *Klebsiella pneumoniae* carbapenemase (KPC)-producing *Klebsiella pneumoniae* (KPC-KP) is increasing in China. The aim of this study was to investigate the molecular epidemiology of KPC-KP in a nosocomial outbreak.

**Methods:**

Fifty-four KPC-KP isolates were consecutively collected between November 2013 and August 2014 during a KPC-KP outbreak in a tertiary care hospital in Beijing, China. Antimicrobial susceptibility was determined by agar dilution. Carbapenemase, extended-spectrum β–lactamase, 16S rRNA methylase, AmpC β-lactamase, and plasmid-mediated quinolone resistance determinants were detected by PCR amplification. The genetic relatedness of isolates was analyzed by pulsed-field gel electrophoresis and multi-locus sequence typing.

**Results:**

All isolates belonged to ST11 except one isolate which was identified as a new sequence type (ST2040). PFGE profile of genomic DNA revealed seven clusters, of which cluster A and C dominated the KPC-KP outbreak and cluster A was replaced by cluster C during the outbreak. PFGE of genomic DNA, S1-PFGE of plasmids, replicon typing, and drug resistant characteristics showed that clonal spread occurred during the outbreak. When compared with isolates within cluster A, all isolates in cluster C harbored *rmtB* and showed higher level of resistance to cefepime, amikacin, tobramycin, and tigecycline.

**Conclusion:**

We reported a nosocomial outbreak of KPC-KP with clonal replacement and a new sequence type (ST2040) of KP. High degree of awareness and surveillance of KPC-KP should be given to avoid potential outbreaks, especially in ICU wards.

## Background

Carbapenem-resistant *Klebsiella pneumoniae* (KP) has spread worldwide and become a major public health threat in health care facilities [1], and the mortality could reach up to 40–50% [2]. According to an antimicrobial resistance surveillance networks in China (CHINET), the rate of carbapenem-resistant KP escalated from 0.7% in 2006 to 10% in 2013 [3], which is mainly due to the rapid dissemination of *Klebsiella pneumoniae* carbapenemase (KPC)-producing *Klebsiella pneumoniae* (KPC-KP) [4]. The *bla*_KPC_ gene can be disseminated by both clonal spread and horizontal plasmid transfer [5]. In China, KPC-KP was firstly identified in 2007 [6]. Since then, this pathogen has been identified in several provinces and ST11 has been demonstrated to be the dominant clone [4, 7–16]. Nosocomial outbreaks of KPC-KP were also described previously [14, 17, 18]. However, clonal replacement of epidemic KPC-KP has not been reported in China or abroad.

Here, we reported a nosocomial outbreak of KPC-KP with clonal replacement in our hospital, which involved 54 consecutive patients and mainly occurred in ICU wards. The drug resistance and epidemiologic features of KPC-KP were also described.

## Methods

### Patients and bacterial isolates

All Carbapenem-resistant KP strains, which were collected between November 2013 and August 2014 in this study, were isolated from different clinical wards of our tertiary care hospital with 1500 beds. Species were identified by the Vitek 2 system, followed by 16S rDNA sequencing. Crude mortality was defined as death during hospitalization. Infection-related mortality was defined as death only as a direct consequence of KPC-KP infection during hospitalization. A KPC-KP outbreak was defined as two or more laboratory-confirmed patients that were temporally related, epidemiologically linked, and infected by the same KP variant.

### Detection of antibiotic resistance genes

Isolates exhibiting resistance to at least one of the carbapenems (imipenem or ertapenem) were evaluated for the presence of *bla*_KPC_ by PCR and sequencing as described previously [19, 20]. All of the KPC-positive isolates were subjected to PCR amplification and sequencing for the presence of carbapenemase genes (*bla*_GES_, *bla*_SME_, *bla*_IMI_, *bla*_BIC_, *bla*_NDM_, *bla*_IMP_, *bla*_VIM_, *bla*_SIM_, and *bla*_SPM_) [19, 20], extended-spectrum β–lactamase genes (*bla*_TEM_, *bla*_SHV_, *bla*_CTX-M_, and *bla*_OXA-48-like_) [21–23], 16S rRNA methylase genes (*armA*, *rmtA*, *rmtB*, *rmtC*, *rmtD*, *rmtE*, and *npmA*) [24, 25], AmpC β-lactamase genes (*bla*_ACC_, *bla*_FOX_, *bla*_MOX_, *bla*_DHA_, *bla*_CIT_ */bla*_SPM_, and *bla*_EBC_) [26], and plasmid-mediated quinolone resistance (PMQR) genes (*qnrA, qnrB, qnrC, qnrS, qepA, acc(6′)-Ib-cr, oqxA,* and *oqxB*) [27].

### Antimicrobial susceptibility testing

Susceptibility tests were performed using the Vitek 2 system and the AST-GN card. MICs of various antimicrobials were determined by agar dilution method and results were interpreted according to the criteria recommended by CLSI (2014) [28].

### Pulsed-field gel electrophoresis (PFGE) analysis

The clonal relatedness of KP isolates were analyzed by PFGE as described previously [29]. Prepared genomic DNA was digested using *Xba*I restriction enzyme on all clinical isolates. The banding patterns were analyzed by the BioNumerics software. The genetic similarity was calculated by dice coefficients and dendrograms were constructed by the unweighted pair group of arithmetic average. The analysis parameters were based on 1.5% tolerance values. Clusters were defined as DNA patterns sharing ≥85% similarity. Plasmid profiling was performed by PFGE of total bacteria genome DNA cut with nuclease S1.

### Multi-locus sequence typing (MLST) analysis

Genotyping was further determined by MLST analysis. Standard DNA amplification and sequencing of seven housekeeping genes (*gapA*, *infB*, *mdh*, *pgi*, *phoE*, *rpoB*, and *tonB*) were performed as described previously [30]. The allele sequences and sequence types (ST) were identified at http://bigsdb.web.pasteur.fr/klebsiella/klebsiella.html.

### PCR-based replicon typing (PBRT)

To determine the plasmid incompatibility groups (F, FIA, FIB, FIC, HI1, HI2, I1-Ic, L/M, N, P, W, T, A/C, K, B/O, X, Y, and FII), PBRT were carried out in all isolates as described previously [31].

### Conjugation experiments

The conjugation experiments were performed by using azide-resistant *E.coli* J53 as the recipient strain. Cultures of donor and recipient cells in logarithmic phase (0.5 ml of each) were added to 4 ml of fresh Luria-Bertani broth and were incubated overnight without shaking. The transconjugants were selected on Mueller-Hinton agar plates containing 100 μg/ml ampicillin and 300 μg/ml sodium-azide. Antibiotic resistance genes and replicon typing were performed as described previously in this study.

### Statistical analysis

All statistical analysis were performed using the SPSS software package version 20.0. Continuous variables (age and Charlson score) were summarized as medians and were compared using non-parametric Mann-Whitney U test. Categorical variables were summarized as percentages and were compared using the pearson chi-square test. A *p*-value of less than 0.05 was considered statistically significant.

## Results

### Description of isolates

A total of 54 non-duplicate KPC-KP isolates were included in this study. All patients, where the 54 isolates were isolated from, were identified as clinical infections based on the isolation of KPC-KP from clinical samples and medical diagnosis established by physicians according to the clinical manifestations and the antibacterial effects. The predominant type of samples from which KPC isolates were isolated was sputum (*n* = 41, 75.9%), followed by bronchoalveolar lavage fluid (*n* = 5, 9.3%), blood (*n* = 4, 7.4%), secretions (*n* = 2, 3.7%), urine (*n* = 1, 1.9%), and peritoneal fluid (*n* = 1, 1.9%). Of note, 77.8% (42/54) of isolates were isolated from patients in two ICU wards (ICU-1, respiratory medicine department; ICU-2, department of critical care medicine), and remaining 22.2% (12/54) of isolates were isolated from other seven medical wards. Specifically, the ICU-1 ward (with 14 beds) and ICU-2 ward (with 12 beds) belonged to two departments and were located in two different buildings with a distance of about 100 m. There were no intercommunications of patients, staffs, and medical instruments between the two ICU wards. Patients in the two ICU wards were scattered in patient rooms or beds.

### Genotypic investigation of resistance

Among the 54 KPC-KP (*bla*_KPC-2_), 1.9% (1/54) carried *bla*_SHV-11_, 48.1% (26/54) carried *bla*_SHV-11_ and *bla*_TEM-1_, and the remaining 50% (27/54) carried *bla*_TEM-1_ *, bla*_SHV_ (*bla*_SHV-1,_ *bla*_SHV-2,_ *bla*_SHV-11_ and *bla*_SHV-12_), and *bla*_CTX_ (*bla*_CTX-M-3_ and *bla*_CTX-M-14_). Among *rmtB*-positive KPC-KP (RP-KP) isolates (*n* = 20), 45.0% (9/20) of isolates carried *qnrS* and 50.0% (10/20) of isolates carried *qnrS* and *acc(6′)-Ib-cr*. However, among *rmtB*-negative KPC-KP (RN-KP) isolates (*n* = 34), only 5.9% (2/34) of isolates carried *acc(6′)-Ib-cr* and 8.8% (3/34) of isolates carried *qnrS* and *acc(6′)-Ib-cr*. Resistance genes encoding class B carbapenemase (*bla*_NDM_, *bla*_SIM_, *bla*_IMP_, *bla*_VIM_, and *bla*_SPM_) and AmpC β–lactamase (*bla*_ACC_, *bla*_FOX_, *bla*_MOX_, *bla*_DHA_, *bla*_CIT_ */bla*_SPM_, and *bla*_EBC_) were not detected in any of the isolates.

### Molecular typing

**Fig. 1:**
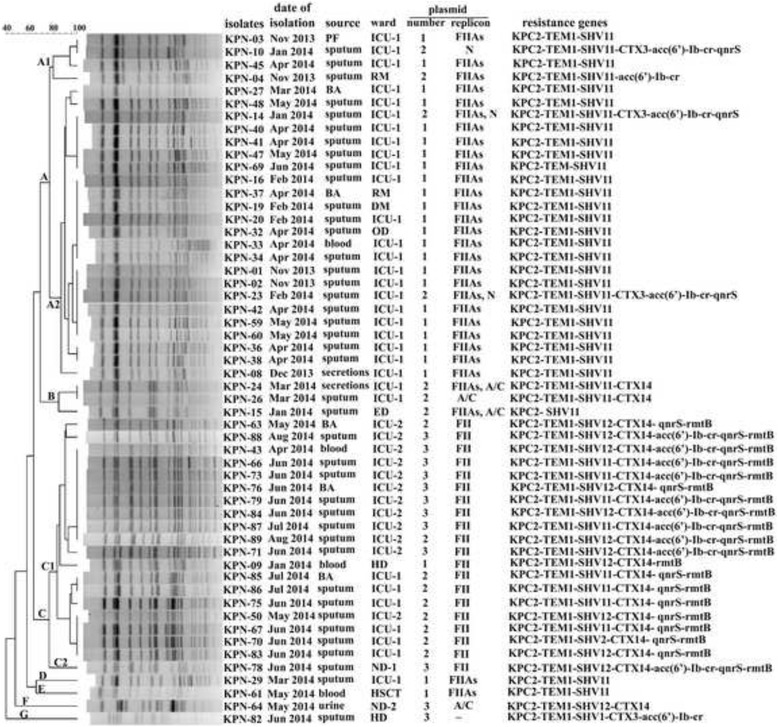
Dendrograms showing the PFGE and epidemiology profiles of 54 KPC-KP isolates. BA, bronchial aspirate; PF, peritoneal fluid; RM, respiratory medicine department; DM, digestive medicine department; OD, oncology department; ED, emergency department; HD, hematology department; ND-1, neurology department; ND-2, nephrology department; HSCT, department of hematopoietic stem cell transplantation; ICU-1, ICU ward of RM; ICU-2, ICU ward of department of critical care medicine; KPC2, KPC-2; TEM1, TEM-1; CTX, CTX-M-

Through KP MLST analysis, 98.1% (53/54) of isolates belonged to ST11 (allelic profile 3–3–1-1-1-1-4). Only one isolate was identified as a new ST (ST2040, allelic profile 3–3–1-1-1-1-326), which only differed from ST11 with a substitution in the *tonB* allele. PFGE identified two major clusters (A, C) among 54 isolates. The cluster A and C consisted of 27 RN-KP isolates and 20 RP-KP isolates, respectively. The other seven RN-KP isolates were classified into the cluster B, D, E, F, and G (Fig. 1).

### Distribution of plasmid replicons

PBRT revealed that 55.6% (30/54) and 37.0% (20/54) of KP isolates contained IncFIIAs and IncFII, respectively. Specifically, 96.3% (26/27) of isolates in cluster A contained IncFIIAs, and 100% (20/20) of isolates in cluster C contained IncFII. After sequencing, the IncFIIAs confirmed in this study was identified as a new subtype of IncFII, which was characterized with low nucleotide identity (90%) when compared with the IncFIIAs replicon of Salmonella virulence plasmids.

### Conjugation experiments

Five predominant isolates (KPN-01, KPN-16, KPN-34, KPN-60, and KPN-69) in cluster A and 11 predominant isolates (KPN-50, KPN-66, KPN-70, KPN-71, KPN-73, KPN-75, KPN-79, KPN-84, KPN-87, KPN-88, and KPN-89) in cluster C were used in conjugation experiments. Of the 16 tested isolates, only one isolate (KPN-69) in cluster A and four isolates (KPN-50, KPN-66, KPN-70, and KPN-88) in cluster C yielded transconjugants. As for replicon type, IncFIIAs and IncFII were confirmed in transconjugants (IncFIIAs: KPN-69; IncFII: KPN-50 and KPN-70). The transconjugant of KPN-69 harbored *bla*_KPC-2_ and *bla*_TEM-1_. The transconjugant of KPN-50 and KPN-70 harbored *bla*_KPC-2_, *bla*_TEM-1_, *bla*_CTX-M_, and *rmtB*. The transconjugant of KPN-66 and KPN-88 harbored *bla*_SHV-11_ (or *bla*_SHV-12_), *bla*_CTX-M_, and *qnrS*.

### Outbreak

**Fig. 2:**
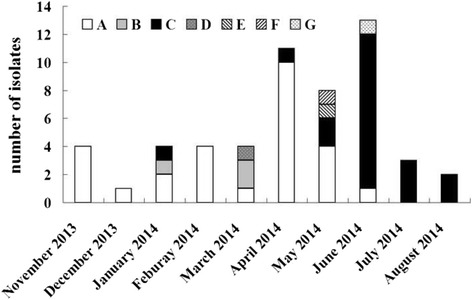
Distribution of KPC-KP isolates by month and PFGE clusters. A to G were designated as PFGE clusters as described in Fig. 1.

The epidemic curve revealed five phases (Fig. 2): phase 1 (Nov 2013 to Mar 2014), fewer cases (< 5 cases per month) mainly caused by cluster A; phase 2 (Apr 2014), a small outbreak (≥ 5 cases per month) caused by cluster A; phase 3 (May 2014), a small outbreak caused by four different clusters; phase 4 (Jun 2014), a small outbreak caused by cluster C; phase 5 (Jul 1014 to Aug 2014), fewer cases mainly caused by cluster C. Of note, cluster A was replaced by cluster C during this KP outbreak. 85.2% (23/27) of isolates in cluster A contained one plasmid with IncFIIs replicon which harbored resistance gene *bla*_KPC_, *bla*_TEM_, and *bla*_SHV_. According to PFGE of genomic DNA, S1-PFGE of plasmids, replicon typing, and drug resistant characteristics (Fig. 1), two main groups in cluster C were suggested to be derived from different origins, of which one group consisted of isolates from the ICU ward of respiratory department (ICU-1) and the other group consisted of strains from the ICU ward of department of critical care medicine (ICU-2). 75% (9/12) of isolates in the former group mainly contained three plasmids with IncFII replicon which harbored *bla*_KPC-2_ *, bla*_TEM-1_ *, bla*_SHV-11_ (or *bla*_SHV-12_), *bla*_CTX-M-14_ *, acc(6′)-Ib-cr, qnrS,* and *rmtB*. However, all strains (*n* = 7) in the latter group contained two plasmids with FII replicon which harbored *bla*_KPC-2_ *, bla*_TEM-1_ *, bla*_SHV-11_ (or *bla*_SHV-12_), *bla*_CTX-M-14_, *qnrS,* and *rmtB*. Specifically, clonal replacement (from cluster A to cluster C) occurred in ICU wards, especially in ICU-1 (Fig. 1).

### Antibiotic susceptibility

Resistance rates in cluster C (RP-KP, *n* = 20) were 100% to ampicillin, piperacillin/tazobactam, cefoxitin, cefepime, aztreonam, imipenem, amikacin, gentamycin, tobramycin, ciprofloxacin, levofloxacin, furantoin, sulfamethoxazole, and 40.0% (8/20) to tigecycline. However, resistance rates in cluster A (RN-KP, *n* = 27) against aforementioned antibiotics were 100% (27/27), 96.3% (26/27), 92.6% (25/27), 22.2% (6/27), 100% (27/27), 100% (27/27), 0 (0/27), 88.9% (24/27), 7.4% (2/27), 100% (27/27), 100% (27/27), 100% (27/27), and 100% (27/27), respectively, as well as 0 (0/27) to tigecycline, which was characterized with significantly lower resistance rates against cefepime (22.2% vs 100%), amikacin (0 vs 100%), tobramycin (7.4% vs 100%), and tigecycline (0 vs 40.0%) when compared with cluster C (*p* < 0.05).

### Mortality discrepancy between clones

Medical records revealed that there were no significant differences in gender (male percentage, 81.5% vs 70.0%; median age, 75 vs 77; median Charlson scores, 6.0 vs 6.0; patient distributions (from ICU wards or transferred from ICU wards), 92.6% vs 95%) in patients between cluster A (*n* = 27) and cluster C (*n* = 20). As for the crude mortality, it was significantly higher in cluster C than those in cluster A (80.0% vs 51.9%, *p* < 0.05). The infection-related mortality in cluster C was higher than those in cluster A although the difference was statistically nonsignificant (25.0% vs 14.8%, *p* > 0.05).

## Discussion

KPC-KP outbreaks have been previously reported in several provinces of China [4, 7–16]. Compared with Western countries, the prevalence of KPC-KP in China is relatively low (2.9%), as described in a 4000-bed tertiary care hospital [32]. However, KPC-KP is an increasing cause for great clinical concern due to its high antibiotic resistance and pathogenicity. In this study, a nosocomial outbreak caused by KPC-KP is described. As shown in Fig. 1, the first clonal replacement of epidemic KPC-KP occurred in ICU wards is confirmed by PFGE of genomic DNA, S1-PFGE of plasmids, replicon typing, and drug resistant characteristics. RP-KP also seems to have a higher dissemination advantage over RN-KP. Although this outbreak was severe in the ICU wards, it was terminated by effective measures, such as hand hygiene intervention, patient isolation, and environmental cleaning. This study suggests that continuous surveillance system and strict infection control measures are necessary and urgent to prevent the spread or outbreak of KPC-KP in health care facilities. It is noteworthy that bacterial culture and susceptibility test require 3–5 days and outbreak might occur during this period of time. Thus, rapid identification of the pathogens is crucial in the active surveillance culture program. On the other hand, considering that colonization frequently precedes infection, early identification of colonized cases might also reduce infections and potential outbreaks.

Although KPC-KP isolates have been identified worldwide, the spread has been proved to be caused by the dominant KP clones (ST258 and ST11). In China, ST11 is the dominant clone of KPC-KP [4]. Some new sequence types of KP have also been frequently described [17, 33]. Here, we reported a new sequence type (ST2040). Compared with the majority of isolates in cluster C (ICU-2), this clone only contains two plasmids (Fig. 1). During the outbreak period, the new ST2040 was only isolated once and didn’t become the epidemic strain. Although resistances are proved to be associated with reduced fitness and virulence of pathogens [34, 35], the clinical resistant KP isolates could survive in those high-density antibiotic environments (health care facilities and day care centers). Thus, as a multi-drug resistant clone, the fitness and pathogenicity of ST2040 might need to be investigated further.

The most frequently encountered ESBLs are TEM-, SHV-, and CTX-M-type β–lactamases. The *bla*_CTX-M_, of which prevalence is increased in KP, is carried mainly by IncFII-type plasmids [36]. In this study, the *bla*_CTX-M_ genes in isolates are also carried by IncFII-type plasmids and could be transferred by conjugation. On the contrary, IncFIIAs plasmids (a new replicon subtype), which harbor *bla*_KPC-2_ *, bla*_TEM-1_, and *bla*_SHV-11_ in isolates of cluster A, seems to be hard to be conjugative.

In this study, 100% (54/54) of isolates are positive for *bla*_SHV_, consistent with the hypothesis that chromosomal *bla*_SHV_ is ubiquitous in KP [37]. The majority of chromosomal SHV, including SHV-1, SHV-11, and its close relatives, are non-ESBLs enzymes. However, most of ESBLs-type SHV enzymes are plasmid-borne. In this study, the prevalence of ESBLs-type SHV enzymes (SHV-2 or SHV-12) and CTX-M enzymes in cluster C is significantly higher than those in cluster A (60% (12/20) vs 0 (0/27, 100% (20/20) vs 11.1% (3/27)). Thus, higher prevalence of cefepime-resistance KP in cluster C might be attributed to the fact that more isolates carry ESBLs enzymes.

PMQR determinants are increasingly reported in KP [14]. In this study, most (86.7%, 13/15) of the *acc(6′)-Ib-cr*-positive isolates also carry *qnrS* (Fig. 1). However, two transconjugants from donor isolates (KPN-66 and KPN-88) co-harbored *qnrS* and *acc(6′)-Ib-cr* only carry *qnrS*, but no *acc(6′)-Ib-cr*. These results suggest that *qnrS* and *acc(6′)-Ib-cr* are located in distinct plasmids, which is different from previous studies that *qnr* alleles are frequently co-expressed with *acc(6′)-Ib-cr* on the same plasmid [27, 38]. Interestingly, all 19 PQMR-determinants-producers in cluster C are also *bla*_CTX-M_ -positive and two transconjugants (KPN-66 and KPN-88) co-carry *qnrS* and *bla*_CTX-M_, indicating a significant correlation between the two determinants among these KP isolates.

Isolates producing 16S rRNA methylase frequently exhibit high level of resistance to almost all clinically important aminoglycosides through methylation of the aminoglycoside-binding site [39]. The resistance mechanism caused by 16S rRNA methylase has been reported in KP, and the prevalence of 16S rRNA methylase genes among clinical KP isolates in China is increasing [40, 41]. KPC-KP isolates are multi-drug resistant, but are usually susceptible to aminoglycosides [42–44]. Aminoglycosides also have a significantly higher microbiologic clearance rate than polymyxin B or tigecycline [45]. Here, 37.0% (20/54) of RP-KP showed strong resistance to amikacin (100%) and tobramycin (100%). Co-production of 16S rRNA methylases in KPC-KP could leave limited therapeutic choices for antibacterial treatment and might be associated with higher mortality in this study.

## Conclusions

In summary, we reported a nosocomial outbreak of KPC-KP with clonal replacement and a new sequence type (ST2040) of KP in our hospital. ST11 is the dominant clone. The outbreak mainly occurred in ICU wards. The clonal spread was responsible for this outbreak. Our study also suggested that a high degree of awareness and surveillance of KPC-KP should be given to avoid potential outbreaks, especially in ICU wards.

## Abbreviations

CLSI: Clinical and laboratory standards institute
ICU: Intensive care unit
KPC: *Klebsiella pneumoniae* carbapenemase
KPC-KP: KPC-producing *Klebsiella pneumoniae*
MIC: Minimum inhibitory concentration
MLST: Multi-locus sequence typing
PBRT: PCR-based replicon typing
PFGE: Pulse field gel electrophoresis
PMQR: Plasmid-mediated quinolone resistance
rDNA: ribosomal deoxyribonucleic acid
rRNA: ribosomal ribonucleic acid
